# Identification of Pre-frailty Sub-Phenotypes in Elderly Using Metabolomics

**DOI:** 10.3389/fphys.2018.01903

**Published:** 2019-01-24

**Authors:** Estelle Pujos-Guillot, Mélanie Pétéra, Jérémie Jacquemin, Delphine Centeno, Bernard Lyan, Ivan Montoliu, Dawid Madej, Barbara Pietruszka, Cristina Fabbri, Aurelia Santoro, Anna Brzozowska, Claudio Franceschi, Blandine Comte

**Affiliations:** ^1^Université Clermont Auvergne, Institut National de la Recherche Agronomique, Unité de Nutrition Humaine, Centre Auvergne Rhône Alpes, Clermont-Ferrand, France; ^2^Université Clermont Auvergne, Institut National de la Recherche Agronomique, Unité de Nutrition Humaine, Plateforme d’Exploration du Métabolisme, MetaboHUB Clermont, Clermont-Ferrand, France; ^3^Nestlé Institute of Health Sciences, Lausanne, Switzerland; ^4^Department of Human Nutrition, Warsaw University of Life Sciences – Szkoła Główna Gospodarstwa Wiejskiego, Warsaw, Poland; ^5^Department of Experimental, Diagnostic and Specialty Medicine, University of Bologna, Bologna, Italy; ^6^Interdepartmental Center “L. Galvani”, University of Bologna, Bologna, Italy; ^7^Institute of Neurological Sciences (IRCCS), Bologna, Italy

**Keywords:** pre-frailty, biomarkers, elderly, sub-phenotypes, gender differences, untargeted metabolomics

## Abstract

Aging is a dynamic process depending on intrinsic and extrinsic factors and its evolution is a continuum of transitions, involving multifaceted processes at multiple levels. It is recognized that frailty and sarcopenia are shared by the major age-related diseases thus contributing to elderly morbidity and mortality. Pre-frailty is still not well understood but it has been associated with global imbalance in several physiological systems, including inflammation, and in nutrition. Due to the complex phenotypes and underlying pathophysiology, the need for robust and multidimensional biomarkers is essential to move toward more personalized care. The objective of the present study was to better characterize the complexity of pre-frailty phenotype using untargeted metabolomics, in order to identify specific biomarkers, and study their stability over time. The approach was based on the NU-AGE project (clinicaltrials.gov, NCT01754012) that regrouped 1,250 free-living elderly people (65–79 y.o., men and women), free of major diseases, recruited within five European centers. Half of the volunteers were randomly assigned to an intervention group (1-year Mediterranean type diet). Presence of frailty was assessed by the criteria proposed by Fried et al. (2001). In this study, a sub-cohort consisting in 212 subjects (pre-frail and non-frail) from the Italian and Polish centers were selected for untargeted serum metabolomics at T0 (baseline) and T1 (follow-up). Univariate statistical analyses were performed to identify discriminant metabolites regarding pre-frailty status. Predictive models were then built using linear logistic regression and ROC curve analyses were used to evaluate multivariate models. Metabolomics enabled to discriminate sub-phenotypes of pre-frailty both at the gender level and depending on the pre-frailty progression and reversibility. The best resulting models included four different metabolites for each gender. They showed very good prediction capacity with AUCs of 0.93 (95% CI = 0.87–1) and 0.94 (95% CI = 0.87–1) for men and women, respectively. Additionally, early and/or predictive markers of pre-frailty were identified for both genders and the gender specific models showed also good performance (three metabolites; AUC = 0.82; 95% CI = 0.72–0.93) for men and very good for women (three metabolites; AUC = 0.92; 95% CI = 0.86–0.99). These results open the door, through multivariate strategies, to a possibility of monitoring the disease progression over time at a very early stage.

## Introduction

The western populations are all aging and the number of people aged over 65 years is continuously increasing. In Europe, the proportion of elderly is expected to be approximately 30% of the population by 2060 (Eurostat, 2011; Beard et al., 2016), with consequences in terms of age-related diseases and disabilities. Aging is a very complex progression involving many biochemical processes in the organism leading to a wide variety of altered biochemical functions, and modifying risks for multiple diseases in a tissue-, organ-, and system-specific manner. In fact, the trajectory of human aging is a continuum of dynamic transitions that involve multifaceted processes, at multiple levels, depending on numerous intrinsic (gender, genes, age, etc.) and extrinsic (nutrition, physical activity, gut microbiota, etc.) factors, and is still far from being fully understood (Franceschi et al., 2000a,b). It is now recognized that chronic, low-grade inflammation or inflammaging, plays a role in the pathogenesis of major age-related diseases (Franceschi et al., 2007; Cevenini et al., 2013) such as frailty and sarcopenia, thus contributing to elderly morbidity and mortality. Frailty is a clinical state characterized by an increased individual’s vulnerability for developing higher dependency and/or mortality when exposed to an environmental stressor. Two main approaches have been developed for defining frailty, either based on numbers of impairments and conditions, combined in a frailty index, or alternatively using a defined specific physical phenotype taking into account five criteria (weight loss, exhaustion, weakness, slowness, and reduced physical activity) (Morley et al., 2013).

As a clinically relevant syndrome that need to be therapeutically addressed, frailty status is usually characterized according to this standardized phenotype, as described by Fried et al. (2001): subjects meeting three or more criteria are classified as frail whereas those with one or two as pre-frail. A recent systematic review by Fernandez-Garrido et al. (2014) reported that the prevalence of pre-frailty can vary in different cohorts, ranging between 35 and 60% in the population aged over 65. Moreover, this review highlighted the necessity to explore the initial phases of frailty development to explore specific involved mechanisms and their potential reversibility. In this context, it is of critical importance to find strategies to better identify these at risk populations and find early and/or predictive biomarkers of age-associated changes to develop more appropriate health care. A critical issue in the field of biomarker (accuracy, specificity and sensitivity) development resides in the intrinsic complexity of frailty, as evidenced by the large spectrum of phenotypes it encompasses (Collino et al., 2013, 2014). Therefore, in order to apprehend the multidimensionality of the aging process, systems biology approaches involving the study of metabolic responses to stimuli with the aim of characterizing the dynamics as well as organ-specific biochemical responses are of great interest (Collino et al., 2011). In this context, metabolomics, consisting of the characterization of a biological system *via* the simultaneous measurement of metabolites, has been shown as a powerful phenotyping tool to molecularly understand (patho)physiology and identify biomarkers of metabolic deviations (Ramautar et al., 2013; Dumas et al., 2014; Lindon and Nicholson, 2014). Indeed, the study of metabolism is of major interest as it reflects molecular physiology downstream of genomics and includes the nutritional and environmental dimension. Moreover, in comparison with single metabolites-based clinical assessments, metabolic signatures provide a direct input into and readout of aging processes, contributing to revealing subtle key metabolic changes and finally to the stratification of health trajectories of elderly (Collino et al., 2017).

The objective of the present study was to better characterize the complexity of pre-frailty phenotype using untargeted metabolomics in order to identify specific biomarkers, and study their stability over time. To fulfill this objective, subjects from the NU-AGE study (Santoro et al., 2014), categorized as not frail or pre-frail (criteria proposed by Fried et al., 2001), were selected and serum samples were analyzed using mass spectrometry metabolomics at T0 (recruitment) and T1 (follow-up, a 1 year after). A multivariate and longitudinal approach was then developed to study sub-phenotypes and their reversibility over time, and finally propose specific circulating biomarkers.

## Materials and Methods

### Study Population

This study is part of the NU-AGE (“New dietary strategies addressing the specific needs of elderly population for a healthy aging in Europe”) project (Santoro et al., 2014). Within this project, a total of 1,250 volunteers, aged 65–79 years and gender balanced, free-living and free of major diseases, categorized as non-frail or pre-frail were enrolled in five European countries (Italy, France, Poland, Netherlands, and United Kingdom). NU-AGE was approved by the Ethics Committee of the coordinator center: the Independent Ethics Committee of the S. Orsola-Malpighi Hospital Bologna (Italy), and by the local/national Ethics Committees of all the other four recruiting centers: the South-East 6 Person Protection Committee (France), the Wageningen University Medical Ethics Committee (Netherlands), the National Research Ethics Committee–East of England (United Kingdom), and the Bioethics Committee of the Polish National Food and Nutrition Institute (Poland). Written informed consent was collected from all participants prior to their inclusion in the study, in accordance with the Declaration of Helsinki. Participants were randomly assigned in two groups: one followed a whole “Mediterranean” type diet (diet group), the other continued its own diet (control group). For each subject, various parameters (clinical, phenotypical, functional, nutritional data) were measured at time zero (T0-baseline) and after 12 months (T1-follow-up) (Berendsen et al., 2014). Regarding the NU-AGE diet, an adherence scoring system was created with cut-off values based on the NU-AGE Food Based Dietary guidelines (Berendsen et al., 2018). The maximum level of intake for several NU-AGE diet components was established based on the country-specific population’s intake distribution (i.e., 85th percentile for sodium and sweets, 100th percentile for whole grains and low-fat meat). In total, the NU-AGE index ranged from 0 to 160 points, ranking participants according to their compliance to the NU-AGE diet.

Frailty was based on the five criteria proposed by Fried et al. (2001) including weight loss, weakness (i.e., poor handgrip strength), self-reported exhaustion, slowness (i.e., slow gait speed), and low physical activity. Weight loss was defined as self-reported unintentional loss (i.e., not due to diet or physical exercise) of ≥4.5 kg in the last 12 months. Handgrip strength was measured three times in the dominant hand using the Scandidact Smedley’s Hand^®^ Dynamometer. Weakness was defined as the average handgrip strength equal or below the sex- and BMI-specific cutoffs provided by Fried et al. (2001). Two questions from the Center for Epidemiologic Studies Depression (CES-D) scale were administered as measures of exhaustion: “I felt that everything I did was an effort” and “I could not get going.” Self-reported exhaustion was present if at least one condition was present for ≥3 days in the past week. Gait speed was measured by asking participants to walk at their usual speed over 4.5 m. Slow gait was defined as walking equal to or above the sex- and height-specific validated cutoffs (Fried et al., 2001). Physical activity was measured with the energy expenditure weekly rate (kcal/week) derived from the modified Minnesota Leisure Time Activity Questionnaire, in which participants were asked on frequency and duration of time spent in 18 activities over the prior 2 weeks (Taylor et al., 1978; Siscovick et al., 1997). Low physical activity was defined as <383 kcal for men or <270 kcal for women, according to sex-specific cutoffs (Fried et al., 2001). Non-frail subjects were defined as for the absence of all the above criteria, pre-frail subjects were defined as for the presence of one or two of the above criteria.

To fulfill the objective of the present work, of better characterizing the complexity of pre-frailty phenotype using metabolomics in order to identify specific biomarkers, and study their stability over time, a sub-cohort was selected for this study. It consists first, in 120 subjects (60 pre-frail, 60 non-frail) randomly selected at baseline among the intervention group, from the Italian and Polish centers. In addition, all the remaining incident subjects shifting their frailty status during the follow-up (*n* = 92) were also selected from the same centers, regardless of their intervention groups. Three sub-groups were then defined: stable subjects, who did not change their frailty status over time (Stable), those who changed their status from pre-frail to non-frail (Improvement), and those who changed from non-frail to pre-frail (Degradation) over 12 months. All these subjects (*n* = 212) were selected for untargeted serum metabolomics at T0 (baseline) and T1 (follow-up).

### Serum Metabolomics Analysis

Untargeted metabolomics was performed following the procedure described in Pujos-Guillot et al. (2017). In brief, sera samples (100 μL) were deproteinized using cold methanol. After evaporation under nitrogen, the dry residues were redissolved in 50/50 (v/v) acetonitrile/water containing 0.1% formic acid. Pooled quality-control samples were prepared by mixing 20 μL from each of the serum samples and prepared similarly. Metabolic profiles were then determined using an ultra-performance liquid chromatography coupled to quadrupole-time-of-flight mass spectrometer (Bruker Impact HD2), equipped with an electrospray source. Separations were carried out using an Acquity HSS T3 column (Waters). Data were acquired in positive and negative ion modes with a scan range from 50 to 1,000 mass-to-charge ratio (*m*/*z*). Samples were randomized within the analytical sequence based on a Williams Latin Square strategy defined according to the main factors of the study: time (T0, T1), gender, country, and pre-frailty status at baseline. The stability of the analytical system was monitored using pooled samples as quality control, injected one time at the beginning of each sequence and then after each set of 10 samples.

### Data Processing and Metabolite Identification

Data were processed under the Galaxy web-based platform Worflow4metabolomics (Giacomoni et al., 2015)^1^, using first XCMS, followed by quality checks and signal drift correction according to the algorithm described by van der Kloet et al. (2009), to yield a data matrix containing retention times, masses and peak intensities that have been corrected for batch effects. These steps include noise filtering, automatic peak detection and chromatographic alignment allowing the appropriate comparison of multiple samples by further processing methods. After statistical analyses, molecular features of interest were searched against an in-house database containing the reference spectra of more than 1,000 authentic standard compounds analyzed in the same analytical conditions. Then, the remaining unknown compounds were identified on the basis of their exact masses which were compared to those registered in Metlin^2^, in the Human Metabolome Database (HMDB ^3^) (Wishart et al., 2007), or in Kyoto Encyclopedia of Genes and Genomes (KEGG) database^4^. Database queries were performed with a mass error of 0.005 Da and a retention time difference of 0.1 min (for the in-house database). Database results were confirmed using appropriate standards when available, isotopic patterns, and mass fragmentation analyses, performed on a Thermo Scientific LTQ Orbitrap Velos hybrid mass spectrometer (Thermo Fisher Scientific, San José, CA, United States) using high resolution, at 100,000 resolving power. For unidentified ions, the number of plausible elemental compositions is restricted to a small number (or uniquely identified) with the support of additional chemical information-i.e., the molecular formula of the parent and knowledge of possible metabolic pathways. Metabolites were classified accordingly to the MSI recommendations (Sumner et al., 2007) concerning the levels of confidence in the identification process: identified (confirmed by standard), putatively annotated (based upon physicochemical properties and/or spectral similarity with public/commercial spectral libraries), putatively characterized compound classes (Supplementary Table S1).

### Statistical Analyses

Characteristics of the studied population and sub-groups were analyzed using Wilcoxon test or Fisher’s exact tests for numerical or categorical data, respectively. Quantitative variables were expressed as means ± SD.

Two-ways ANOVA was applied on metabolomics data at T0 and T1, respectively, with BH correction, to assess the effects of phenotype (pre-frail vs. non-frail) for each gender, with country as cofactor. These analyses were performed using the Galaxy web-service Worflow4metabolomics, first in the whole study population, and then in each sub-groups of interest [(i) stable subjects, (ii) subjects who improved their frailty status, and (iii) subjects who worsened their status]. Calculation of a *p*-value for each feature allowed identifying the significant ones (adjusted *p*-values ≤ 0.05). The log_2_ mean ratios of ion intensities of pre-frail vs. non-frail were calculated to represent the fold change for the molecular features. The most significant ions (corrected *p* < 0.01) were then selected to build prediction models using multivariate logistic regression. In a first step, the variables that were the most correlated to others (paired-wise Pearson correlations) were eliminated one by one to reduce redundant information. In a second step, multi-collinearity among remaining variables were addressed using Variance Inflation Factor and proportion of variance. Finally, the subset of variables was introduced in a multiple logistic regression model, and the final model was determined using the Akaike Information Criterion (AIC) values in a stepwise method. Because of the relatively small sample size in sub-groups, a cross-validation technique was used to ensure that the logistic regression models were robust. The dataset was iteratively randomly divided into a training set to fit the model and a test set to evaluate it (the two subsets having the same size), and 100 iterations were performed. The prediction model performance was evaluated using a confusion matrix, error rates, and area under ROC curves (AUC) (Xia et al., 2013) [R package “pROC” (Robin et al., 2011)] with a CI estimated with the DeLong’s method (DeLong et al., 1988).

## Results

### Study Population

**FIGURE 1:**
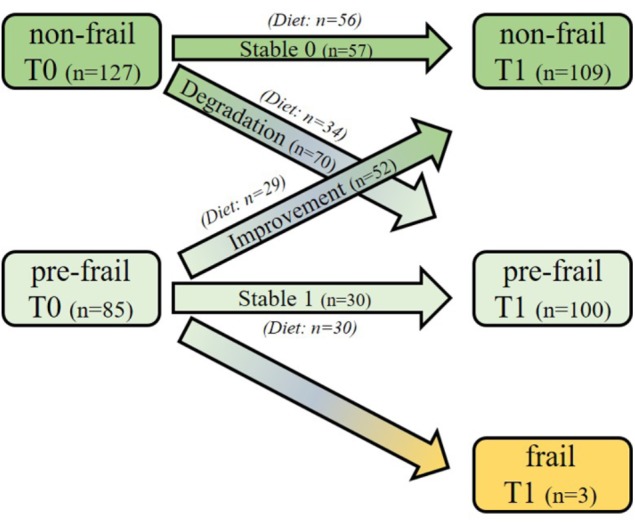
Study design and frailty status evolution of the subjects over the 1-year period. One hundred and twenty subjects (60 pre-frail and 60 non-frail) were first randomly selected at baseline among the intervention group from the Italian and Polish centers. In addition, all the remaining incident subjects shifting their frailty status during the follow-up (*n* = 92; *n* = 67 non-frail and *n* = 25 pre-frail) were also selected from the same centers, regardless of their intervention groups. Three sub-groups were defined: *Stable subjects*, who did not change their frailty status over time [Stable 0 (*n* = 57; 56 of them followed the NU-AGE diet); Stable 1 (*n* = 30, all of them followed the diet)], those who changed their status from pre-frail to non-frail [*Improvement* (*n* = 52, 29 of them were in the NU-AGE diet group)] and those who changed from non-frail to pre-frail [*Degradation* (*n* = 70, 34 of them followed the NU-AGE diet)] over 12 months.

Figure 1 presents the evolution of the subject status over time for the selected sub-cohort as well as their belonging to the intervention groups: over the 212 selected subjects, 85 were pre-frail and 127 were non-frail at baseline. Among the pre-frail subjects, 52 individuals improved their frailty status, and 70 non-frail subjects became pre-frail during the follow-up period. Moreover, three pre-frail subjects became frail.

Table 1 presents the characteristics of the studied groups at baseline. Because of gender differences, all statistical analyses were performed independently in each gender, with country as cofactor. In terms of frailty criteria, weakness was found to be the most prevalent (around 50% for each gender). Thirteen % of men cases and 21% of women presented two criteria, respectively. Except for whole body lean mass (corrected *p*-value < 2 × 10^-16^) and handgrip test measure (<3 × 10^-5^), no clinical parameters, neither anthropometric nor nutritional ones were found significant (Supplementary Table S2).

**Table 1:** Characteristics of the study population at baseline (T0) stratified by gender and pre-frailty status.

	Men (*n* = 91)			Women (*n* = 121)		
	Non-frail	Pre-frail	Corrected *p*-value (BH)	Non-frail	Pre-frail	Corrected *p*-value (BH)
Number of subjects	60	31	–	67	54	–
Age (years)	71.2 ± 3.9	72.7 ± 3.6	0.36	70.6 ± 3.8	71.7 ± 3.7	0.66
Frailty criteria (*n*, %)						
Weakness	–	16 (51.6)		–	27 (50.9)	
Shrinking	–	7 (22.6)		–	15 (9.4)	
Endurance	–	6 (19.3)		–	20 (37.7)	
Low activity	–	5 (16.1)		–	10 (18.9)	
Slowness	–	1 (3.2)		–	2 (3.8)	
SPPB score	11.6 ± 0.8	11.3 ± 0.8	0.47	11.3 ± 1.2	10.8 ± 1.5	0.33
Hand grip test measure (kg)	39.9 ± 6.2	32.9 ± 8.2	**4.2 × 10^-4^**	24.6 ± 3.8	20.8 ± 5.6	**5.1 × 10^-4^**
Gait speed (sec)	3.6 ± 0.6	3.9 ± 1.0	0.44	3.8 ± 0.6	4.2 ± 1.1	0.13
Body Mass Index (BMI, kg/m^2^)	27.3 ± 4.0	28.2 ± 3.6	0.70	27.0 ± 3.9	28.5 ± 3.9	0.34
Whole body weight (kg)	81.5 ± 14.1	83.6 ± 13.8	0.78	68.5 ± 10.7	70.9 ± 12.3 (*n* = 53)	0.68
Body fat mass (kg)	24.7 ± 8.9	26.4 ± 8.6	0.74	28.4 ± 8.2	30.6 ± 8.8 (*n* = 53)	0.68
Body lean mass (kg)	53.8 ± 6.5	54.1 ± 6.3	0.91	37.9 ± 3.7	38.2 ± 4.6 (*n* = 53)	0.97

*Data are presented as means ± SD. Bold: significant *p*-values (≤0.05) in ANOVA, with Benjamini-Hochberg (BH) correction, to assess the effects of phenotype (pre-frail vs. non-frail). Gait speed: time (s) needed to walk for 4.57 m. SPPB, Short Physical Performance Battery.*

### Pre-frailty Sub-Phenotypes at Baseline

**FIGURE 2:**
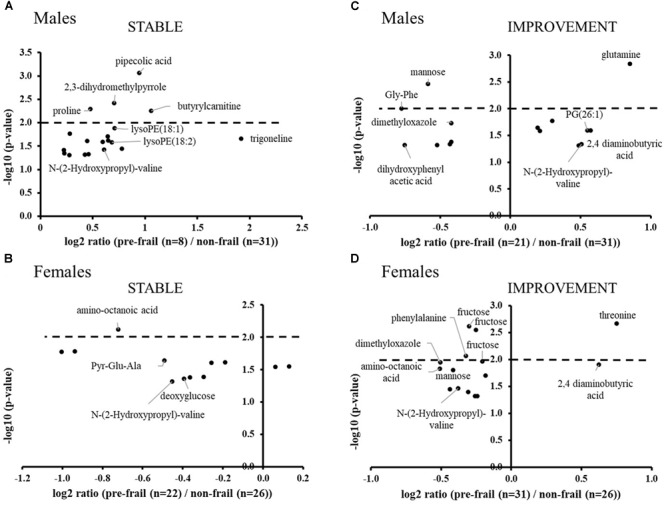
Volcano plots of significant ions for pre-frailty status at baseline in the stable population (**A**: males and **B**: females) and in subjects who improved their frailty status (**C**: males and **D**: females). ANOVA with country as cofactor; *p*-values corrected for multiple testing (Benjamini-Hochberg) < 0.05. The dot lines represent the *p*-value cut-off used for the logistic regression analyses (*p*-values < 10^-2^).

Untargeted metabolomics performed on serum samples collected at baseline revealed significant ions between pre-frail and non-frail subjects after ANOVA [with country as cofactor; *p*-values corrected for multiple testing (BH) < 0.05], with large differences depending on gender and pre-frailty groups (Stable or Improvement). Figure 2 shows the significance and amplitude of the modulated metabolites in male and female subjects. Ten to twenty ions were found significant in each sub-group with relative high *p*-values (corrected *p* > 10^-3^) and moderate fold changes [log_2_ ratios (pre-frail/non-frail) between -1 and 2]. Results highlighted specific metabolic signatures in the different sub-groups with only one marker in common [*N*-(2-hydroxypropyl)-valine].

In the stable population, pipecolic acid, 2,3-dihydromethylpyrrole, proline, butyrylcarnitine, and lysoPEs were found significantly upregulated in pre-frail men, whereas in women, most of the significant metabolites were found down regulated with amino-octanoic acid being the most significant one (corrected *p* = 7.5 × 10^-3^). In this group, weakness and endurance were the most prevalent criteria: 75% and 68%; 25% and 32% in males and females, respectively. Moreover, the handgrip test measure was found to be significantly decreased for pre-frail subjects (log_2_ ratio ∼-0.5; corrected *p*-value < 2 × 10^-2^ for both genders).

In the group of subjects who will improve their frailty status (Improvement), the most significant modulated metabolites are amino acids and derivatives, as well as sugars. Regarding phenotypic data, weakness, shrinking and low activity criteria were found the most prevalent (38, 33, and 24% of the pre-frail population, respectively) for men of this group, whereas women were found to be less heterogeneous with the major presence of weakness and endurance (40%).

### Prediction of Pre-frailty Status at Baseline

**FIGURE 3:**
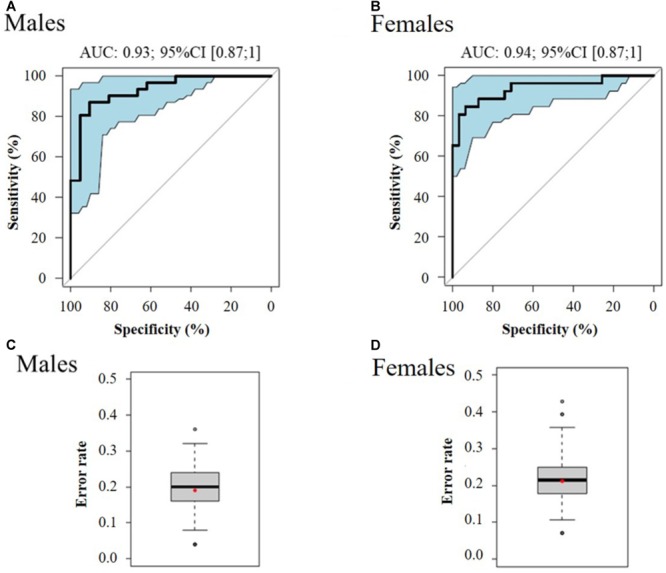
ROC curve of the best multivariate prediction models of pre-frailty status at baseline (**A**: males and **B**: females) and box-plots of the error rate corresponding to the 100 cross-validated models (**C**: males and **D**: females). Data from serum metabolomics at baseline, of subjects who improved their pre-frailty status (*n* = 52) *versus* stable non-frail individuals (*n* = 57).

In order to classify the pre-frail subjects who improved their status over time from the non-frail ones at baseline, we performed multivariate logistic regressions considering significant metabolites with a corrected *p*-value < 10^-2^. The best-reduced models included four metabolites for each gender, namely glutamine, Gly-Phe, dimethyloxazole, and mannose for men; and threonine, fructose, mannose, and *N*-(2-hydroxypropyl)-valine for women (Table 2). To evaluate the performance of these multivariate models, the ROC and AUCs were computed using the initial training and the cross-validation sets. Figures 3A,B show the sensitivity and specificity values defining the aforementioned areas under the ROC of the two best multivariate models. Results showed very good prediction capacity with AUCs of 0.93 (95% CI 0.87–1) and 0.94 (95% CI 0.87–1), similar sensitivity, specificity, and error rates (15 and 14%) for men and women, respectively (Table 3 and Figures 3C,D: box-plots of the error rate corresponding to the 100 cross-validated models). It is interesting to note that when trying to stratify the stable subjects using these models, the predictive performance was poor (18% and 33% of misclassified subjects for men and women, respectively), especially regarding stable pre-frail male subjects (87% misclassification).

**Table 2:** Regression models for the prediction of pre-frailty status at baseline from serum metabolomics data.

Model	Variables	Coefficient β	Standard error	Pr(>| z|)	Odd ratios (95% CI)
Men	Constant offset β0	5.9	2.3	0.010	
	Glutamine	6.6 × 10^-4^	2.6 × 10^-4^	0.011	1.0007 (1.0002–1.0012)
	Gly-Phe	–1.8 × 10^-3^	7.6 × 10^-4^	0.018	0.998 (0.997–0.9997)
	Dimethyloxazole	–1.8 × 10^-3^	8.3 × 10^-4^	0.028	0.998 (0.997–0.9998)
	Mannose	–1.3 × 10^-3^	5.2 × 10^-4^	0.012	0.999 (0.998–0.9997)
Women	Constant offset β0	14.7	5.0	0.003	
	Threonine	4.1 × 10^-4^	1.5 × 10^-4^	0.007	1.0004 (1.0001–1.0007)
	Fructose	–1.7 × 10^-3^	5.9 × 10^-4^	0.004	0.998 (0.997–0.9994)
	Mannose	–1.3 × 10^-3^	5.0 × 10^-4^	0.009	0.999 (0.998–0.9997)
	*N*-(2-hydroxypropyl)-valine	–1.4 × 10^-3^	6.8 × 10^-4^	0.033	0.999 (0.997–0.9999)

*Formal equation of logistic regression model is written as log(π) = β0 + β1X1 + β02X2 + … + βkXk, where π is the probability of proportion of pre-frail in group, and Xi is metabolite intensity.*

**Table 3:** Performance of the best multivariate regression models for the prediction of pre-frailty status at baseline from serum metabolomics data.

Model	AUC (95% CI)	Sensibility (%)	Specificity (%)	Error rate (%)
Men	0.93 (0.87-1)	81%	87%	15%
Women	0.94 (0.87-1)	85%	87%	14%

*AUC, area under the ROC curve.*

In order to evaluate the modulation of these metabolites over time in this population of changers (Improvement), similar analyses were performed at T1, when all subjects were assessed as non-frail. Some of the metabolites were still significant: 2,4-dimethylamino butyric acid (*p* = 0.01), Gly-Phe (*p* = 0.006), for men; and 2,4-dimethylamino butyric acid (*p* = 0.008), threonine (*p* = 0.003), and dimethyloxazole (*p* = 0.009) for women. Moreover, some of them were also modulated by the diet: dimethyloxazole (*p* = 0.05) in men and fructose (*p* = 0.04) for women after ANOVA diet vs. control (*p*-values corrected for multiple testing).

### Replication/Validation at Follow-Up, in Changers Who Worsened Their Status

**FIGURE 4:**
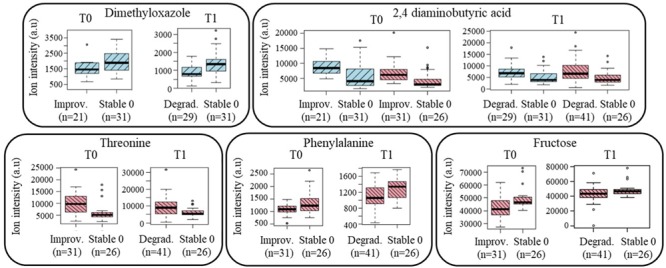
Boxplots of the ‘light’ pre-frailty biomarkers discovered at baseline in subjects who will improve their status (Improvement; *n* = 52) and valid at follow-up in subjects who just worsened it (Degradation; *n* = 70). Blue color: men’s data; red color: women’s data (a.u, arbitrary units).

The initial approach of discovering pre-frailty discriminant metabolites was replicated in the sub-group of subjects who became pre-frail over time, with the analyses of their plasma samples at T1 *versus* stable non-frail individuals. Figure 4 shows the boxplots of the metabolites that were previously identified at baseline in subjects who showed a reversible pre-frailty status and also found valid in this sub-population (Degradation), just after the appearance of pre-frailty: 2,4-diaminobutyric acid for both genders, dimethyloxazole for men, and threonine, phenylalanine and fructose for women. These metabolites can therefore be considered of major interest to be included in a molecular signature of a ‘light’ pre-frailty phenotype. Some others were found to be specific of the degradation only in women: glutamic acid, threonine, lactose, alanine, and one remaining unknown (C_3_H_8_NO_4_Mg). Regarding the frailty criteria, endurance was found the most prevalent in both genders (59 and 54% for men and women, respectively), with shrinking present at 21% in men and weakness at 34% in women.

### Early Signature and Prediction of Evolution Toward Pre-frailty

**FIGURE 5:**
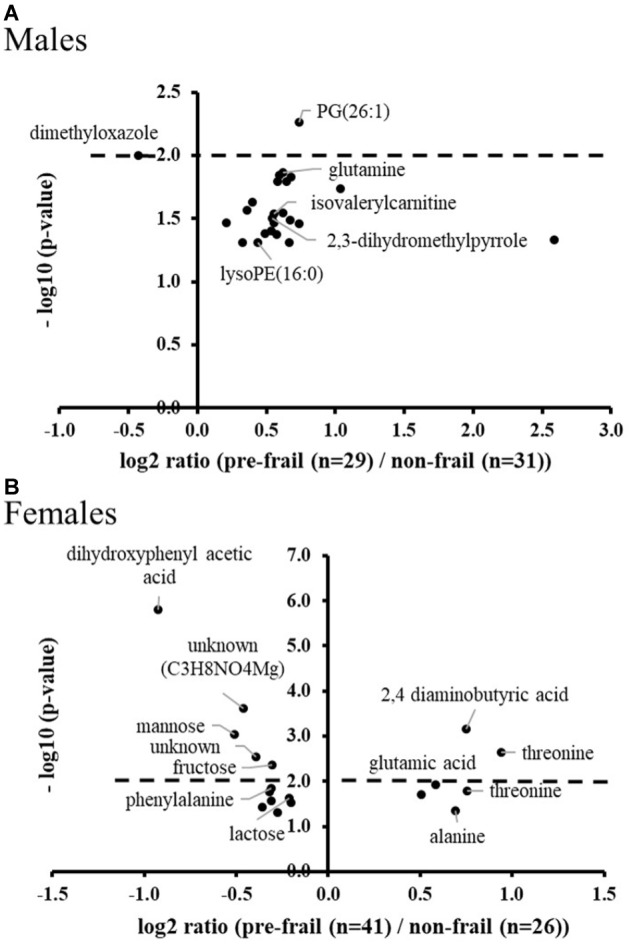
Volcano plots of early pre-frailty markers. Significant ions at baseline in subjects (**A**: males and **B**: females) who worsened their frailty status over time (Degradation) *versus* stable non-frail subjects. ANOVA with country as cofactor; *p*-values corrected for multiple testing (Benjamini-Hochberg) < 0.05. The dot lines represent the *p*-value cut-off used for the logistic regression analyses (*p*-values < 10^-2^).

The population of changers who will worsen their status during the follow-up was then analyzed at T0 in comparison to stable non-frail subjects, to discover potential early and/or predictive pre-frailty discriminant metabolites. Around 20 significant ions were identified from metabolomic profiles with no common ones between men and women. Among the most significant metabolites (corrected *p* < 10^-2^), PG (26:1) and dimethyloxazole were found modulated in men; dihydroxyphenyl acetic acid, 2,4-aminobutyric acid, mannose, fructose and threonine (Figure 5) in women. It is important to note that some of these early discriminant metabolites of pre-frailty were already previously identified as discriminant of the ‘light’ pre-frailty phenotype: 2,4-aminobutyric acid, dimethyloxazole, threonine and fructose.

**FIGURE 6:**
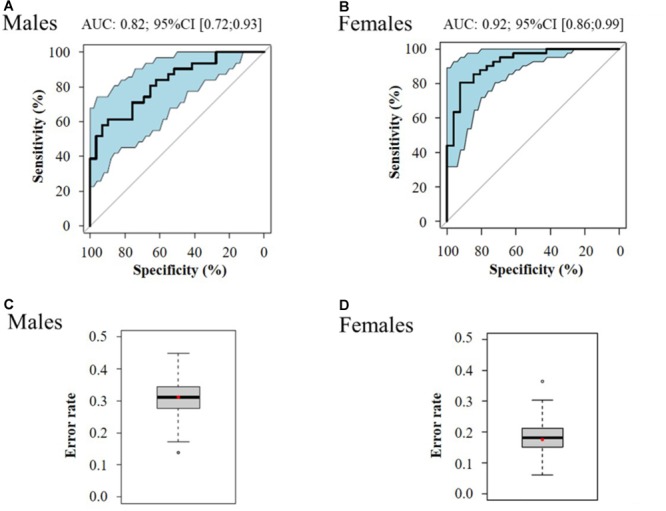
ROC curve of the best multivariate prediction models of evolution toward pre-frailty (**A**: males and **B**: females) and box-plots of the error rate corresponding to the 100 cross-validated models (**C**: males and **D**: females).

Multivariate logistic regressions performed considering the most significant metabolites (corrected *p* < 10^-2^), allowed obtaining predictive models of evolution toward pre-frailty. The best models include dimethyloxazole, glutamine and isovalerylcarnitine for men, and dihydroxyphenyl acetic acid, threonine, and mannose for women (Table 4). The ROC and AUCs were computed using the initial training and the cross-validation sets. Figures 6A,B show the sensitivity and specificity values defining the aforementioned areas under the ROC of the two best multivariate models. Prediction performance of these gender specific molecular signatures was found to be good for men with AUC of 0.82 (95% CI 0.72–0.93, 30% error rate) and very good for females with AUC of 0.92 (95% CI 0.86–0.99; 16% error rate) (Table 5 and Figures 6C,D: box-plots of the error rate corresponding to the 100 cross-validated models).

**Table 4:** Best multivariate regression models for the prediction of evolution toward pre-frailty from serum metabolomics data at baseline.

Model	Variables	Coefficient β	Standard error	Pr(>|z|)	Odd ratios (95% CI)
Men	Constant offset β0	–0.2	1.1	0.85	
	Dimethyloxazole	–1.5 × 10^-3^	5.5 × 10^-4^	0.007	0.998 (0.997–0.9996)
	Glutamine	3.7 × 10^-4^	1.8 × 10^-4^	0.038	1.0004 (1.000–1.0007)
	Isovalerylcarnitine	6.3 × 10^-4^	2.7 × 10^-4^	0.018	1.0006 (1.000–1.0011)
Women	Constant offset β0	4.2	1.5	0.005	
	Dihydroxyphenyl acetic acid	–9.0 × 10^-4^	2.7 × 10^-4^	0.001	0.999 (0.998–0.9996)
	Threonine	2.9 × 10^-4^	1.0 × 10^-4^	0.004	1.0003 (1.000–1.0005)
	Mannose	–1.2 × 10^-3^	4.8 × 10^-4^	0.010	0.999 (0.998–0.9997)

*Formal equation of logistic regression model is written as log(π) = β0 + β1X1 + β02X2 + … + βkXk, where π is the probability of proportion of pre-frail in group, and Xi is metabolite intensity.*

**Table 5:** Performance of regression models for the prediction of the evolution toward pre-frailty from serum metabolomics data at baseline.

Model	AUC (95% CI)	Sensibility (%)	Specificity (%)	Error rate (%)
Men	0.82 (0.72–0.93)	71%	83%	30%
Women	0.92 (0.86–0.99)	83%	84%	16%

*AUC, area under the ROC curve.*

## Discussion

The results of this study showed for the first time, that untargeted metabolomics enabled the identification of circulating pre-frailty molecular signatures in serum. Even if few studies have evaluated physiological differences associated with frailty (Carcaillon et al., 2012; Fazelzadeh et al., 2016; Sebastiani et al., 2017) most of them did not consider its preclinical stage. Therefore, there is a need of identification of blood pre-frailty biomarkers that would be very useful for both prevention and comprehension of the disease etiology (Fernandez-Garrido et al., 2014).

Some of the makers identified in the present study were already reported in frailty. Plasma levels of proline, butyrylcarnitine, and isovalerylcarnitine were associated with muscle metabolites in frailty. Moreover, glutamine, glutamic acid, and isovalerylcarnitine were found as markers of frailty in the muscle. In another hand, pipecolic acid was found as modulated by training in healthy older adults (Fazelzadeh et al., 2016). Phosphoethanolamine species, the second most abundant membrane phospholipid in mammals, have been identified as modulators of inflammation and apoptosis (Gibellini and Smith, 2010), but little is known about specific molecular species. However, the presence of lysoPEs, recognized as oxidized products, suggest an increased inflammatory environment in pre-frailty (Gonzalez-Covarrubias, 2013). To the best of our knowledge, carbohydrates or peptides have never been described in pre-frailty. Nevertheless, these compound classes have been found as modified with old age (Lawton et al., 2008). Moreover, it is interesting to note the importance of some of the plasma glycoproteins (i.e., haptoglobin, transferrin, and fibrinogen) as markers of pre-frailty (Shamsi et al., 2012; Miura and Endo, 2016). Some hormone biomarkers of frailty such as testosterone, DHEA, and cortisol, shown as unchanged in pre-frail individuals (Fernandez-Garrido et al., 2014), were also not found discriminant in the present study.

It is important to highlight that among the discriminant metabolites, several of them are markers of status, linked with nutrition, namely dimethyloxazole, dihydromethylpyrrole, trigonelline, and dihydroxyphenyl acetic acid. In fact, metabolomics integrates information regarding food components and their metabolism and consequently adresses the complex issue of diet and health interaction. Markers of nutrition associated with frailty have already been described (Fernandez-Garrido et al., 2014). Nevertheless, to the best of our knowledge, those observed in the present study focused on pre-frailty have not been reported so far. Correlations were performed between serum nutrient levels and these metabolites and no significant relationships were observed. No relationship was neither observed with the NU-AGE diet compliance score. Finally, vitamin D, 25-hydroxyvitamin D, already described as markers of pre-frailty (Fernandez-Garrido et al., 2014) were not found discriminant in this study, but no difference was observed in serum nutrients between pre-frail and non-frail at T0 and T1 in all groups.

This study also revealed pre-frailty sub-phenotypes, depending on gender and severity of the status. Regarding gender differences, these results are in line with previous publications (Walston and Fried, 1999; Carcaillon et al., 2012; Gale et al., 2013), highlighting differences in prevalence and markers, suggesting different underlying biological mechanisms. Concerning frailty characteristics of the studied subjects, regardless to gender, weakness was the most prevalent criteria of pre-frailty as it has been reported in several studies (Xue et al., 2008; Drey et al., 2011; Smit et al., 2013). However, stable individuals were found more advanced in the pathology, with significant differences in handgrip test measures between pre-frail and non-frail subjects for both genders, in opposite with what was observed in ‘light’ pre-frailty groups.

Given the complexity of pre-frailty sub-phenotypes related to pathophysiological processes, one single biomarker cannot adequately reflect these conditions. Therefore, it has been shown that multivariate biomarker discovery should be performed to be able to identify specific signatures relevant to distinguish the stage and the severity of the conditions (Calvani et al., 2017). In the present study, our exploratory approach allowed identifying a set of markers of ‘light pre-frailty.’ The identified biomarkers were replicated and validated on different subjects of the sub-cohort (Improvement/Degradation) with some common biomarkers found, both for a reversible pre-frailty status and for an early stage of pre-frailty just after its appearance. The resulting multivariate models showed very good predictive capacity, with both high sensitivity and specificity. Different studies evaluated diagnostic test accuracy for identifying frailty in older population (Clegg et al., 2015) and underlined limited specificity resulting in many false-positive results and therefore limiting the use of these instruments. In the present study focusing on pre-frailty, functional variables related to Fried criteria were even not significant for this ‘light pre-frail’ status, showing the interest of the development of more specific markers. However, diagnostic cut-offs for these multivariate markers remain to be identified, after replication in other cohorts and validation using quantitative assays. This perspective is of particular importance to define their validity within the timeframe of the pre-frailty development.

In addition to its capability of disease stratification, metabolomics has also been shown to be powerful to discover early/predictive biomarkers as it can reveal very subtle differences. Therefore, it has been used in different prospective studies to predict the development of other chronic diseases (Kenny et al., 2010; Shah et al., 2012; Wang-Sattler et al., 2012; Pujos-Guillot et al., 2017). To the best of our knowledge, this study is the first to report gender specific, early signatures, able to predict the evolution toward pre-frailty within 1 year. Moreover, some of these early discriminant metabolites were found to be still relevant for classification of ‘light pre-frail’ phenotype after its clinical appearance. This result opens the door to longitudinal analysis of individual time trajectories that could help to detect early deviations of health status.

## Conclusion

In this study, metabolomics allowed discriminating metabolic sub-phenotypes of pre-frailty both at the gender level and depending on the pre-frailty progression and reversibility, with the identification of specific circulating discriminant metabolites. From these results, we were able to build multivariate predictive models for pre-frailty occurrence as well as for 1-year prevalence. Beyond this discovery step, these molecular signatures need to be qualified in large and diverse pre-frail populations in order to study their robustness and precision. Such multivariate strategies based on molecular signatures should then enable the monitoring of the disease progression at an early stage.

## Author Contributions

EP-G and BC contributed to the conception and design of the current work, data analyses, data interpretation, and drafted the manuscript. ClF conceived, designed, initiated, and directed the NU-AGE project. AS coordinated NU-AGE data collection across centers. DC and BL performed the metabolomics analyses. MP and JJ were in charge of statistical analyses. IM revised data analyses and interpretation. DM, BP, CrF, and AB substantially contributed to the clinical, phenotypical, functional, and nutritional data collection by acquiring or processing data. All authors contributed to the interpretation of data, critically revised and approved the final version of the manuscript.

## Conflict of Interest Statement

The authors declare that the research was conducted in the absence of any commercial or financial relationships that could be construed as a potential conflict of interest.

## Footnotes

1 http://workflow4metabolomics.org/

2 https://metlin.scripps.edu

3 www.hmdb.ca

4 http://www.genome.jp/kegg/

## Abbreviations

ANOVA: analysis of variance
AUC: area under the curve
BH: Benjamini-Hochberg
BMI: body mass index
CI: confidence interval
ROC: receiver operating characteristic
SPPB: Short Physical Performance Battery
