# Supplementary material for: Identification of Pre-frailty Sub-Phenotypes in Elderly Using Metabolomics
Source: Front Physiol. 2019 Jan 24;9:1903. doi: 10.3389/fphys.2018.01903 (PMC6353829; doi:10.3389/fphys.2018.01903)
Supplement: Supplementary file 1 [file Table_1.DOCX]

Supplementary Material

**Identification of pre-frailty sub-phenotypes in elderly using metabolomics**

**Estelle Pujos-Guillot^1,2^, Mélanie Pétéra^2^, Jérémie Jacquemin^2^, Delphine Centeno^2^, Bernard Lyan^2^, Ivan Montoliu^3^, Dawid Madej^4^, Barbara Pietruszka^4^, Cristina Fabbri^5^, Aurelia Santoro^5,6^, Anna Brzozowska^4^, Claudio Franceschi^7^, Blandine Comte^1*^**

* **Correspondence**: Blandine Comte; blandine.comte@inra.fr

# Supplementary Tables

**Table 1S:** Summary of identifications of serum metabolites.

^1^Identified compounds confirmed with authentic standards (level 1, (Sumner et al., 2007)), ^2^Putatively annotated compounds (level 2, (Sumner et al., 2007)), ^3^Putatively characterized compound (level 3, (Sumner et al., 2007)).

| **Metabolic Pathway** | **Name** | **Variable label** | **Annotation** | **Retention time (min)** | **Measured mass (*m*/*z*)** |
| --- | --- | --- | --- | --- | --- |
| Amino acid metabolism | Glutamine^1^ | M151T50 | [M+Na-H_2_O]^+^ | 0.83 | 151.04774 |
|  | 2,4 diaminobutyric acid^1^ | M101T50 | [M+H-H_2_O]^+^ | 0.84 | 101.07092 |
|  | Threonine^1^ | M120T51 | [M+H]^+^ | 0.85 | 120.06543 |
|  |  | M74T51 | [M+H-CH_2_O_2_]^+^ | 0.85 | 74.06007 |
|  | Phenylalanine^1^ | M131T359 | [M+H-H_2_O-NH_3_]^+^ | 5.99 | 131.04920 |
|  | Pipecolic acid^2^ | M130T70 | [M+H]^+^ | 1.16 | 130.08639 |
|  | Proline^1^ | M116T57 | [M+H]^+^ | 0.95 | 116.0703 |
|  | Amino-octanoic acid^3^ | M160T491 | C_8_H_18_O_2_N^+^ | 8.19 | 160.13318 |
|  | Glutamic acid^1^ | M130T50 | [M+H-H_2_O]^+^ | 0.84 | 130.04982 |
|  | Alanine^1^ | M90T51 | [M+H]^+^ | 0.84 | 90.05485 |
| Carbohydrate metabolism | Mannose^1^ | M198T53 | [M+NH_4_]^+^ | 0.88 | 198.09741 |
|  | Fructose^1^ | M383T52 | [2M+Na]^+^ | 0.87 | 383.11607 |
|  |  | M384T52 | ^13^C[2M+Na]^+^ | 0.87 | 384.11950 |
|  |  | M443T52 | [2M+2Na+K-2H]^+^ | 0.87 | 443.07214 |
|  | Lactose^1^ | M325T52 | [M+Na-NaOH]^+^ | 0.87 | 325.11298 |
|  | Deoxyglucose^1^ | M187T58 | [M+H]^+^ | 0.98 | 187.05772 |
| Lipid metabolism | Butyrylcarnitine^1^ | M232T412 | [M+H]^+^ | 6.87 | 232.15438 |
|  | Isovalerylcarnitine^1^ | M246T473 | [M+H]^+^ | 7.88 | 246.17003 |
|  | LysoPE(16:0)^1^ | M454T904 | [M+H]^+^ | 15.06 | 454.29240 |
|  | lysoPE(18:1)^2^ | M480T927 | [M+H]^+^ | 15.45 | 480.30998 |
|  | lysoPE(18:2)^2^ | M478T874 | [M+H]^+^ | 14.57 | 478.29360 |
|  | PG(26:1)^3^ | M636T625 | C_32_H_61_O_10_P^+^ | 10.41 | 636.39859 |
| Peptides | N-(2-Hydroxypropyl)-valine^2^ | M176T61 | [M+H]^+^ | 1.01 | 176.12791 |
|  | Gly-Phe^1^ | M223T435 | [M+H]^+^ | 7.26 | 223.10662 |
|  | Pyr-Glu-Ala^3^ | M351T58 | C_13_H_20_N_4_NaO_6_^+^ | 0.97 | 351.12618 |
| Food metabolites | Dimethyloxazole^2^ | M98T371 | [M+H]^+^ | 6.18 | 98.06028 |
|  | 2,3-dihydromethylpyrrole^3^ | M84T71 | C_5_H_9_N | 1.18 | 84.08110 |
|  | trigoneline^1^ | M138T56 | [M+H]^+^ | 0.94 | 138.05439 |
|  | Dihydroxyphenyl acetic acid^3^ | M169T41 | C_8_H_8_O_4_^+^ | 0.69 | 169.04605 |
| Unknown | Unknown | M146T41 | C_3_H_8_NO_4_Mg^+^ | 0.68 | 146.02986 |
|  | Unknown | M610T45 |  | 0.75 | 609.96090 |

**
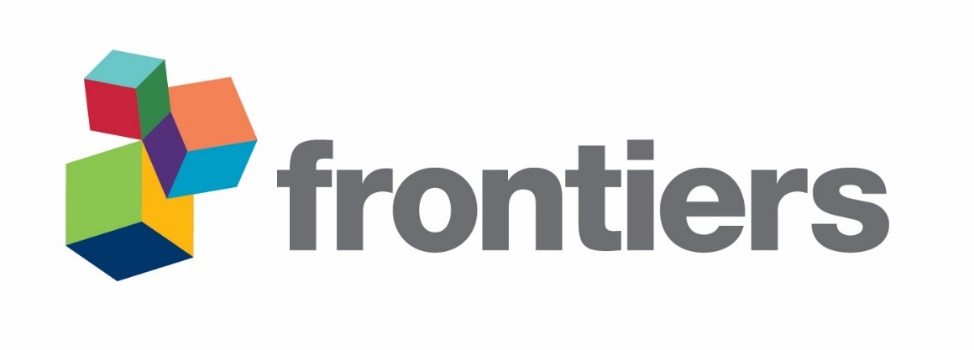
**
