# Supplementary material for: Identification of Pre-frailty Sub-Phenotypes in Elderly Using Metabolomics
Source: Front Physiol. 2019 Jan 24;9:1903. doi: 10.3389/fphys.2018.01903 (PMC6353829; doi:10.3389/fphys.2018.01903)
Supplement: Supplementary file 2 [file Table_2.docx]

Supplementary Material

**Identification of pre-frailty sub-phenotypes in elderly using metabolomics**

**Estelle Pujos-Guillot^1,2^, Mélanie Pétéra^2^, Jérémie Jacquemin^2^, Delphine Centeno^2^, Bernard Lyan^2^, Ivan Montoliu^3^, Dawid Madej^4^, Barbara Pietruszka^4^, Cristina Fabbri^5^, Aurelia Santoro^5,6^, Anna Brzozowska^4^, Claudio Franceschi^7^, Blandine Comte^1*^**

* **Correspondence**: Blandine Comte; blandine.comte@inra.fr

# Supplementary Tables

**Table 2S**: Characteristics of the study population at baseline.

Data are presented as means ± SD. Bold: significant p-values (≤ 0.05) in ANOVA, with Benjamini-Hochberg (BH) correction, to assess the effects of phenotype (pre-frail *vs* non-frail).

|  | Men (n=91) | | | Women (n=121) | | |
| --- | --- | --- | --- | --- | --- | --- |
|  | Non-frail | Pre-frail | corrected  p-value (BH) | Non-frail | Pre-frail | corrected  p-value (BH) |
| Number of individuals | 60 | 31 | - | 67 | 54 | - |
| PASE score | 138.7±55.8 | 144.0±52.1 | 0.85 | 115.1±43.1 | 111.1±47.1 (n=52) | 0.97 |
| *Anthropometric parameters* | | | | | | |
| Height (cm) | 171.5±6.1 | 171.0±5.8 | 0.85 | 158.5±5.2 | 157.0±6.3 | 0.68 |
| Waist circumference (cm) | 98.5±11.8 | 99.5±10.4 | 0.85 | 87.7±9.8 | 90.7±10.8 (n=53) | 0.66 |
| Waist/hip ratio | 0.98±0.07 | 0.96±0.05 | 0.67 | 0.85±0.06 | 0.86±0.06 (n=52) | 0.81 |
| *Blood parameters* | | | | | | |
| Total cholesterol (mM) | 4.87±0.91 | 4.55±0.96 | 0.44 | 5.41±0.92 | 5.36±0.88 | 0.98 |
| HDL-cholesterol (mM) | 1.32±0.40 | 1.22±0.37 | 0.67 | 1.66±0.50 | 1.67±0.51 | 1.00 |
| LDL-cholesterol (mM) | 2.98±0.87 | 2.83±0.78 | 0.74 | 3.19±0.77 | 3.15±0.85 | 0.98 |
| Triglycerides (mM) | 1.24±0.56 | 1.13±0.37 | 0.70 | 1.24±0.66 | 1.19±0.44 | 0.97 |
| Fasting glycemia (mM) | 5.90±0.67 (n=59) | 6.12±1.30 | 0.70 | 5.67±0.62 (n=66) | 5.75±0.73 | 0.97 |
| Fasting insulin (mUI/L) | 11.62±8.40 | 11.10±7.18 | 0.87 | 10.10±6.10 | 11.46±6.23 | 0.68 |
| HOMA-IR | 3.14±2.50 (n=59) | 3.17±2.58 | 0.96 | 2.61±1.72 (n=66) | 2.98±1.72 | 0.69 |
| *Intake data* | | | | | | |
| Energy (kcal) | 1965±474 | 1928±475 | 0.85 | 1573±343 | 1625±404 | 0.85 |
| Proteins (g) | 79.6±21.8 | 82.4±25.6 | 0.82 | 65.6±14.8 | 67.3±16.2 | 0.97 |
| Proteins, animal origin (g) | 48.3±22.0 | 48.9±19.7 | 0.94 | 38.7±13.7 | 40.3±14.7 | 0.85 |
| Proteins, plant origin (g) | 26.7±8.6 | 29.4±10.9 | 0.62 | 22.5±7.0 | 22.1±7.4 | 0.98 |
| Carbohydrates (g) | 249±75 | 253±77 | 0.87 | 202±46 | 209±62 | 0.85 |
| Fat (g) | 72.4±21.5 | 69.1±17.4 | 0.74 | 59.6±17.1 | 62.6±19.2 | 0.79 |
| Saturated fat (g) | 24.6±9.3 | 24.0±8.5 | 0.85 | 20.3±7.9 | 22.4±9.6 | 0.68 |
| Vitamin A (µg) | 1163±1110 | 1331±776 | 0.74 | 1201±998 | 1195±957 | 1.00 |
| Vitamin B12 (µg) | 5.4±5.1 | 6.0±4.6 | 0.85 | 4.5±3.7 | 4.2±3.3 | 0.97 |
| Vitamin C (mg) | 120±81 | 137±73 | 0.70 | 113±48 | 131±63 | 0.60 |
| Vitamin D (µg) | 3.86±3.48 | 4.36±3.51 | 0.76 | 3.18±1.90 | 3.21±2.60 | 1.00 |
|  |  |  |  |  |  |  |
| NU-AGE compliance | 78.8±14.5 | 87.2±15.3 | 0.12 | 84.7±15.3 | 86.6±17.9 | 0.97 |

Sumner, L.W., Amberg, A., Barrett, D., Beale, M.H., Beger, R., Daykin, C.A., et al. (2007). *Metabolomics* 3(3)**,** 211-221. doi: 10.1007/s11306-007-0082-2.

**
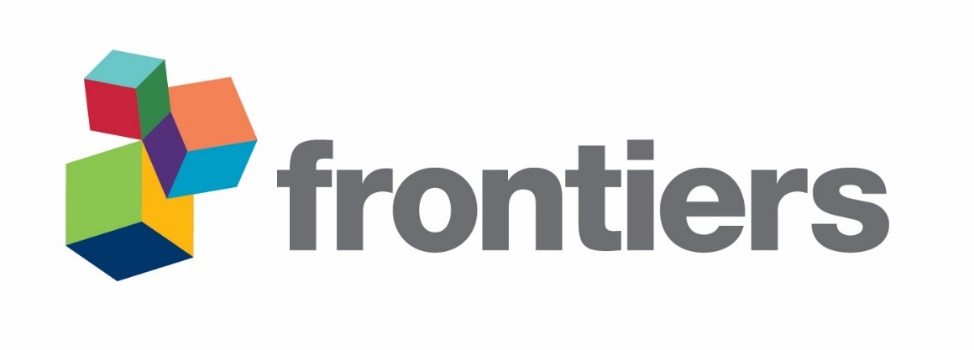
**
